# Identification of the Genes That Contribute to Lactate Utilization in *Helicobacter pylori*

**DOI:** 10.1371/journal.pone.0103506

**Published:** 2014-07-31

**Authors:** Shun Iwatani, Hiroyuki Nagashima, Rita Reddy, Seiji Shiota, David Y. Graham, Yoshio Yamaoka

**Affiliations:** 1 Department of Medicine-Gastroenterology, Baylor College of Medicine and Michael E. DeBakey Veterans Affairs Medical Center, Houston, Texas, United States of America; 2 Department of Environmental and Preventive Medicine, Oita University Faculty of Medicine, Oita, Japan; Institut Pasteur Paris, France

## Abstract

*Helicobacter pylori* are Gram-negative, spiral-shaped microaerophilic bacteria etiologically related to gastric cancer. Lactate utilization has been implicated although no corresponding genes have been identified in the *H. pylori* genome. Here, we report that gene products of *hp0137–0139* (*lldEFG*), *hp0140–0141* (*lctP*), and *hp1222* (*dld*) contribute to D- and L-lactate utilization in *H. pylori*. The three-gene unit *hp0137–0139* in *H. pylori* 26695 encodes L-lactate dehydrogenase (LDH) that catalyzes the conversion of lactate to pyruvate in an NAD-dependent manner. Isogenic mutants of these genes were unable to grow on L-lactate-dependent medium. The *hp1222* gene product functions as an NAD-independent D-LDH and also contributes to the oxidation of L-lactate; the isogenic mutant of this gene failed to grow on D-lactate-dependent medium. The parallel genes *hp0140–0141* encode two nearly identical lactate permeases (LctP) that promote uptake of both D- and L-lactate. Interestingly an alternate route must also exist for lactate transport as the knockout of genes did not completely prevent growth on D- or L-lactate. Gene expression levels of *hp0137–0139* and *hp1222* were not enhanced by lactate as the carbon source. Expression of *hp0140–0141* was slightly suppressed in the presence of L-lactate but not D-lactate. This study identified the genes contributing to the lactate utilization and demonstrated the ability of *H. pylori* to utilize both D- and L-lactate.

## Introduction

Many aerobic and anaerobic bacteria are able to utilize D- and/or L-lactate as carbon and electron sources for their metabolism and respiration [1]. *Helicobacter pylori* are Gram-negative, spiral-shaped microaerophilic bacterium associated with chronic gastric infection leading to gastritis, peptic ulcer, and other gastric disorders [2]. *H. pylori* are found within the gastric mucus layer and attached to cells lining the stomach (i.e., sites where they obtain some growth benefits from the host). For example, in vitro studies have demonstrated that nutrients including L-lactic acid (L-lactate) released by gastric epithelial cells enhanced the growth of *H. pylori* [3]–[6]. However, a role for lactate in the metabolism of *H. pylori* remains unclear as two early genome sequences of *H. pylori* failed to identify a homologous gene to L-lactate dehydrogenase (*lldD*) [7]–[9]. Lactate utilization genes are well characterized in *Escherichia coli*, where a specific permease (*lctP*), L-lactate dehydrogenase (*lctD*, latter termed as *lldD*), and a dedicated transcriptional reglator (*lctR*) are present as an overlapping cluster arranged as an operon [10], [11]. The *lldD*-encoding L-lactate dehydrogenase (L-LDH) catalyzes the oxidation of L-lactate using molecular oxygen to produce pyruvate and hydrogen peroxide [12]. LldD shows homologies with several flavin mononucleotide (FMN)-dependent enzymes in both prokaryotes and eukaryotes, and orthologs have been found in both Gram-negative and Gram-positive bacteria.

The whole genome sequences of *H. pylori* strains 26695 and J99 revealed a putative flavoprotein D-lactate dehydrogenase (Dld; HP1222/JHP1143) and putative lactate permeases (LctP; HP0140–0141/JHP0128–0129) [7], [9]. More recent studies using a comparative genomics approach have also uncovered some of the genes required in bacterial utilization of lactate. Studies by Pinchuk *et al*. in *Shewanella oneidensis* MR-1 revealed new genes encoding another type of D- and L-LDH (Dld-II, LldEFG) potentially involved in D- and L-lactate utilization of a variety of bacteria [13], and there is increasing evidence that the orthologous genes (*lldEFG*/*lutABC* or *dld-II*) are involved in D- and L-lactate utilization in other bacteria [14]–[17]. Since L-lactate is the major stereoisomer of lactate found in human-associated niche, it seems likely that it would be involved in *H. pylori* metabolism.

In this study, we investigated the gene cluster, *hp0137–0139*, that shows homologies with *lldEFG* described in recent reports [13]. The functions of *hp1222* and *hp0140/0141* were also characterized with respect to D- and L-lactate utilization by *H. pylori*.

## Materials and Methods

### Bacterial strains, media, and culture conditions

The bacterial strains and plasmids used in this study are shown in Table 1. All *H. pylori* strains and *Campylobacter jejuni* ATCC33292 were routinely grown on brain-heart infusion (BHI, Difco) agar plate supplemented with 7% (v/v) defibrinated horse blood, or in Brucella broth (Difco) containing 5% (v/v) fetal bovine serum (FBS, Gibco) with rotatory shaking at 100 rpm. All *H. pylori* cultures were grown at 37°C under microaerophilic conditions [5% (v/v) oxygen, 10% (v/v) carbon dioxide and 85% (v/v) nitrogen] in gas evacuation jars. *E. coli* strains were grown on Luria-Bertani (LB, Difco) agar plates or in LB broth at 37°C with shaking at 200 rpm. As necessary, bacterial cultures were supplemented with appropriate antibiotics; chloramphenicol, 4 µg mL^−1^ for *H. pylori* and 34 µg mL^−1^ for *E. coli*; kanamycin, 20 µg mL^−1^ for *H. pylori* and 50 µg mL^−1^ for *E. coli*.

**Table 1:** Bacterial strains and plasmids used in this study.

Strains or plasmids	Characteristics or genotypes*	Reference or source
***Helicobacter pylori***		
26695	*H. pylori* wild-type strain, parental strain for mutants in this study	ATCC[9]
Δ140	*hp0140–0141* disrupted derivative of 26695, Cm^r^	This study
Δ138	*hp0137–0139* disrupted derivative of 26695, Cm^r^	This study
Δ1222	*hp1222* disrupted derivative of 26695, Kan^r^	This study
Δ138/Δ1222	*hp0137–0139* and *hp1222* disrupted derivative of 26695, Cm^r^, Kan^r^	This study
J99	*H. pylori* wild-type strain	[7]
NCTC 11637	*H. pylori* wild-type strain, identical to CCUG 17874	[39]
G27	*H. pylori* wild-type strain	[40]
TN2GF4	*H. pylori* wild-type strain	[41]
SS1 (Sydney strain)	*H. pylori* wild-type strain (mouse-adapted)	[42]
*Campylobacter jejuni* ATCC 33292	*C. jejuni* wild-type strain	ATCC
*Escherichia coli* DH5α	Host strain used for plasmid construction	Takara
**Plasmids**		
pCR Blunt II TOPO	Blunt-end cloning vector; Zeo^r^, Kan^r^	Invitrogen
pGH32	pT7Blue vector with chloramphenicol resistance gene (*cat*); Amp^r^, Cm^r^	lab collection
pGH84	pT7Blue vector with kanamycin resistance gene (*aph3*); Amp^r^, Kan^r^	lab collection
pSI01	TOPO vector with 4.8 kb fragment containing *hp0140*-*hp0141*	This study
pSI02	pSI01 derivative with *hp0140*-*hp0141*::*cat*	This study
pSI03	TOPO vector with 2.8 kb fragment containing *hp1222*.	This study
pSI04	pSI03 derivative with *hp1222*::*aph3*	This study
pSI05	TOPO vector with 2.8 kb fragment containing *hp0137*-*hp0139*	This study
pSI06	pSI05 derivative with *hp0137–0139*::*cat*	This study

*Cm^r^, chloramphenicol resistance; Kan^r^, kanamycin resistance; Zeo^r^, zeocin resistance; Amp^r^, ampicillin resistance.

### Growth assay in conditioned media

For the exchange of primary carbon source, conditioned medium (BB) was prepared by replicating the composition of Brucella broth but without dextrose (D-glucose); namely, it contained 2.0% (w/v) polypeptone peptone (Difco), 0.2% (w/v) yeast extract (Difco), 0.5% (w/v) sodium chloride (Sigma-Aldrich) and 0.01% (w/v) sodium bisulfite (Sigma-Aldrich) as basal components. BB medium was alternatively supplemented with 5 mM D-glucose (Sigma-Aldrich) (BBG), 10 mM L-lactate (Sigma-Aldrich) (BBL) or 10 mM D-lactate (Sigma-Aldrich) (BBD), and was supplemented with 5% (v/v) FBS before use. Minimal conditioned medium (PP) was prepared by reducing or eliminating the components of BB medium and contained 0.5% (w/v) polypeptone peptone, 0.5% (w/v) sodium chloride and 0.01% (w/v) sodium bisulfite as minimal components. PP medium was alternatively supplemented with 5 mM D-glucose (PPG), 10 mM L-lactate (PPL), or 10 mM D-lactate (PPD). In addition, supplemental FBS was reduced to 1% (v/v) to minimize the concentration of glucose and L-LDH that are potentially contained in FBS.

For growth assay, bacterial strains from frozen stocks were grown on a BHI blood agar plates for 48–72 h. The recovered strains were then pre-cultured in 5 mL Brucella-FBS broth for 24 h. The exponentially growing cells were harvested and resuspended in normal saline to adjust the optical density at 600 nm (OD_600_) to 0.2, and 100 µL of the resuspended cells were inoculated into 10 mL of culture medium. Bacterial growth was periodically monitored by measuring the OD_600._ All reported values are the means and standard deviation (SD) of at least three independent experiments.

### Genetic manipulation and transformation

Cloning and other genetic manipulations were performed according to the methods described by Sambrook & Russell [18]. Extraction of bacterial chromosomal DNA was performed using QIAamp DNA mini kit (Qiagen). Plasmid DNAs were purified from *E. coli* strains by using QIAprep spin miniprep kit (Qiagen). Synthetic oligonucleotide primers were obtained from Sigma-Aldrich and shown in Table S1. DNA-sequencing reactions were performed by GENEWIZ, Inc. All the transformations of both *H. pylori* and *E. coli* strains were carried out by electroporation method following the protocol of Gene Pulser II (Bio-Rad Laboratories).

### Construction of *H. pylori* 26695 isogenic mutants

Target gene disruption with the insertion of antibiotic resistance markers was performed using a PCR approach. A 4.8-kb gene fragment containing *hp0140–0141* (3.3 kb) and flanking regions (5′-732 bp, 3′-769 bp) was amplified from *H. pylori* 26695 genomic DNA by PCR using Lct1-F and Lct2-R primers and cloned into pCR Blunt II TOPO vector to produce pSI01. After the sequence was confirmed, pSI01 was used as template for inverse PCR using Lct3-F and Lct4-R primers to amplify the complete plasmid excluding the two genes. The corresponding PCR fragments were blunt-end ligated with non-polar chloramphenicol resistance gene (*cat*) from pGH32 (lab collection) and introduced into *E. coli* DH5α. The resulting plasmid pSI02, harboring *hp0140–0141*::*cat* cassette, was purified and the sequence was confirmed. Similarly, the *hp1222* gene fragment (2.8 kb) was amplified by using hp1222-F0/hp1222-R0 primers and cloned into TOPO vector (pSI03). Inverse PCR with hp1222-F1/hp1222-R1 amplified the complete plasmid excluding the central 1.3-kb part of *hp1222*. The amplified fragment was blunt-end ligated with non-polar kanamycin resistance gene (*aph3*) from pGH84 (lab collection) to generate a plasmid containing *hp1222*::*aph3* cassette (pSI04). Another 2.8-kb gene fragment harboring *hp0137–0139* was amplified by PCR with hp139-F0/hp137-R0 primers. The primer HP0139-F0 was designed to have one nucleotide substitution (A>T) so as to introduce a termination codon in 5′ region of *hp0139*. The PCR fragment was then cloned into TOPO vector (pSI05), and pSI05 was used in inverse PCR with hp137-F0/hp139-R0 primers to amplify the complete plasmid excluding the first codon of *hp0137*, whole region of *hp0138*, and the last codon of *hp0139*. The PCR fragment was ligated with the *cat* fragment to produce pSI06, which has the *hp0137–0139*::*cat* cassette to inactivate all the three genes. Finally, the plasmids pSI02, pSI04, and pSI06 were used to amplify each gene knockout cassette with the initial primer sets (Lct1-F/Lct2-R, hp1222-F0/hp1222-R0, and hp139-F0/hp137-R0), and the PCR fragments were used to transform *H. pylori* 26695 by electroporation method. Appropriate antibiotic resistant colonies were screened and the mutations were confirmed by sequencing of those genomic DNAs.

### RNA extraction and qRT-PCR

To verify the non-polar effects of those mutations and for further quantification, total RNAs were extracted from *H. pylori* strains using RNeasy mini kit (Qiagen) that were exponentially growing in appropriate liquid media. The extracted RNAs were then treated with DNase I (Invitrogen) to remove contaminating genomic DNA, and reverse-transcribed with SuperScript III (Invitrogen) to synthesize the first-strand cDNA. In conventional RT-PCR, the primer sets hp138-qF/hp138-qR, hp140-qF/hp140-qR, hp1222-qF/hp1222-qR, and Hp16S-F/Hp16S-R were used to amplify 100–150 bp of each *hp0138*, *hp0140*, *hp1222*, and 16S rRNA gene. Real-time quantitative PCRs were performed in 7300 Real-Time PCR System (Applied Biosystems) using Taqman-designed primers and probes (Life Technologies) and the data were analyzed by the comparative Ct (ΔΔCt) method. All the quantifications of target genes were performed in triplicate and were shown as the mean value ± SD.

### D- and L-lactate import/consumption assay

Exponentially growing *H. pylori* cells (OD_600_; 0.15–0.2) were harvested from 30 mL of Brucella-FBS broth. The cell pellets were washed once with phosphate buffer saline (PBS, pH 7.4) and resuspended in PP medium to adjust the OD_600_ to 0.4. The bacterial suspension was then mixed with an equal amount of PPL or PPD medium, resulting in the mixture containing live bacterial cells (OD_600_; 0.2) as well as 5 mM L-lactate or D-lactate. The mixed media were incubated at 37°C in microaerophilic conditions with rotatory shaking at 100 rpm. Culture supernatants were collected after 3, 6, 9 hours of incubation and stored at −80°C until required. The concentration of lactate in supernatants was measured by colorimetric assays using L-lactate assay kit (Sigma-Aldrich) and D-lactate assay kit (Eton Bioscience) according to the manufacture’s instructions. All the data represent means ± SD of three independent experiments.

### D- and L-lactate dehydrogenase activity assay

Exponentially growing *H. pylori* cells (OD_600_; 0.15–0.2) were harvested from 30 mL of Brucella-FBS broth. The cell pellets were washed twice with ice cold 10 mM Tris-HCl (pH 8.0), and resuspended in the same buffer to adjust the OD_600_ to 0.5. Bacterial cells were disrupted by sonication (VirSonic 60; 10 W output, 5×10 sec with 1 min interval on ice) and cell extracts were obtained after removal of debris by centrifugation at 13,000 rpm, 4°C for 10 min. Total protein concentration of cell extract was determined by the BCA method (Pierce) before the use for enzyme activity assays. NAD-independent D- and L-lactate dehydrogenase activities (D- and L-iLDH) were measured by the previously described colorimetric assay (MTT assay) using 5 mM D- or L-lactate as an electron donor and a mixture of phenazine methosulfate (PMS) and 3-(4,5-dimethylthiazolyl-2)-2,5-diphenyltetrazolium bromide (MTT) as artificial acceptors [13], [19], [20]. Briefly, 5 µL of the cell extract (1.5–2.0 µg protein) was added to 200 µL of reaction mixture containing 50 mM Tris-HCl (pH 8.0), 0.5% (v/v) Triton X-100, 120 µg/mL PMS, 60 µg/mL MTT, and 5 mM D- or L-lactate. The reaction was started by the addition of substrate, and the increase in absorbance at 570 nm was monitored by a microplate spectrophotometer (Epoch, BioTek) with an automated correction of absorbance value to reflect a path length of 1 cm (Gen5, BioTek). Specific activities were calculated using an MTT extinction coefficient of 17 mM^−1^cm^−1^ at 570 nm, and normalized by the total protein concentration in cell extract. NAD-dependent L-lactate dehydrogenase activity (L-nLDH) was measured by using a colorimetric assay kit (Sigma-Aldrich, MAK066). In this assay, L-nLDH reduced NAD to NADH along with the conversion of L-lactate into pyruvate which was specifically detected by colorimetric (450 nm) assay and was calibrated based on enclosed NADH standards. One unit of LDH activity is defined as the amount of enzyme that catalyzes the conversion of lactate into pyruvate to generate 1.0 µmole of NADH per minute at 37°C.

## Results

### Inactivation of the candidate genes for lactate permease and D- and L-LDH in *H. pylori* 26695

**Figure 1:**
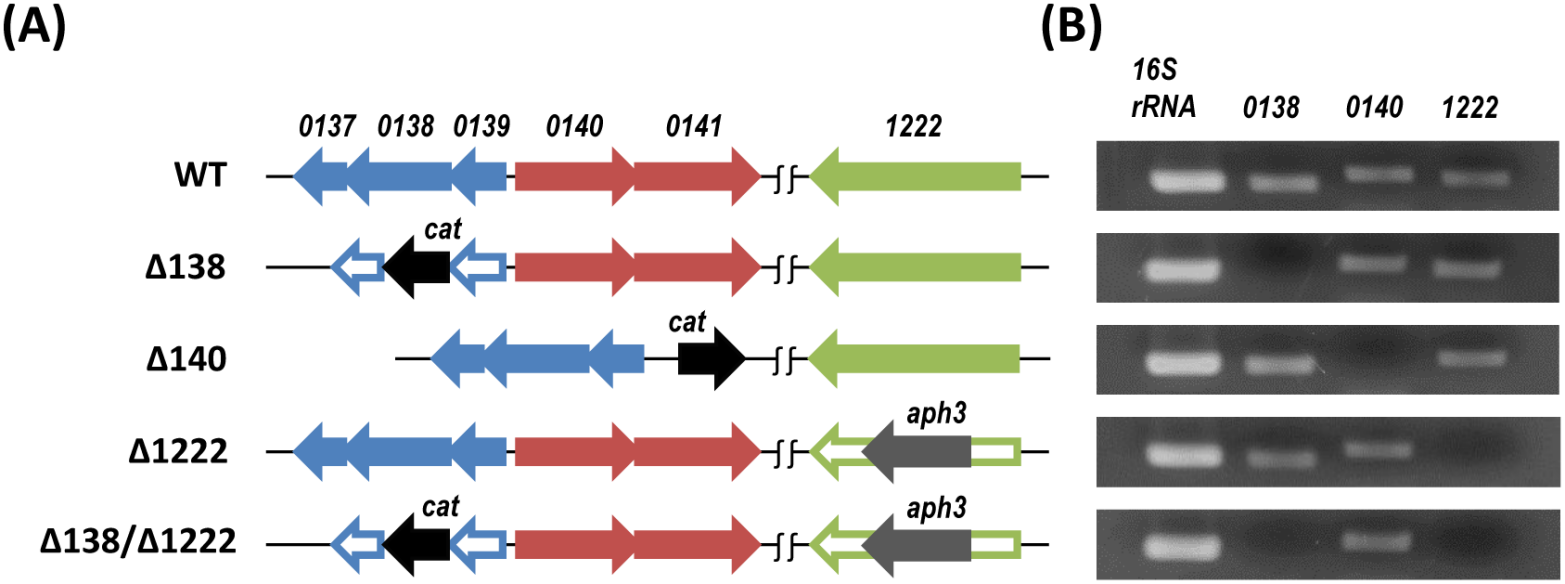
Gene organization of *H. pylori* 26695 wild type and isogenic mutants (A), and RT-PCR products of relevant genes (B). Colored arrows indicate intact genes, and void arrows indicate inactivated genes. *cat*; chloramphenicol resistance gene, *aph3*; kanamycin resistance gene. RT-PCR products were obtained from 30 cycles of amplification.

The *H. pylori* 26695 genome contains three-gene unit *hp0137–0138–0139* at 230-bp upstream of *hp0140–0141* genes that are seemingly duplicated and tandemly located in opposite direction, while *hp1222* is located at a distance away from the former five genes (Fig. 1A). It was previously reported that the gene cluster *lldEFG* functioned as a unit, and the disruption of each of three genes resulted in the loss of the L-LDH ability [15], [17]. Thus, all the genes of *hp0137–0139* were disrupted collectively in the mutant Δ138 just as described in Materials and Methods. The gene products HP0140 and HP0141 share 72% identity with 85% positive residues suggesting each product plays much the same role in function; thereby, we entirely disrupted the two genes in the mutant Δ140. The *hp1222* gene was disrupted with the insertion of another antibiotic resistance marker (*aph3*) so that the single gene mutant (Δ1222) as well as the multiple genes mutant (Δ138/Δ1222) could be generated. The RT-PCR products amplified from total RNAs of 26695 mutants clearly demonstrated the missing transcripts of target genes (Fig. 1B).

### HP0137–0139 are required for growth on L-lactate, while HP1222 is required for growth on D-lactate

**Figure 2:**
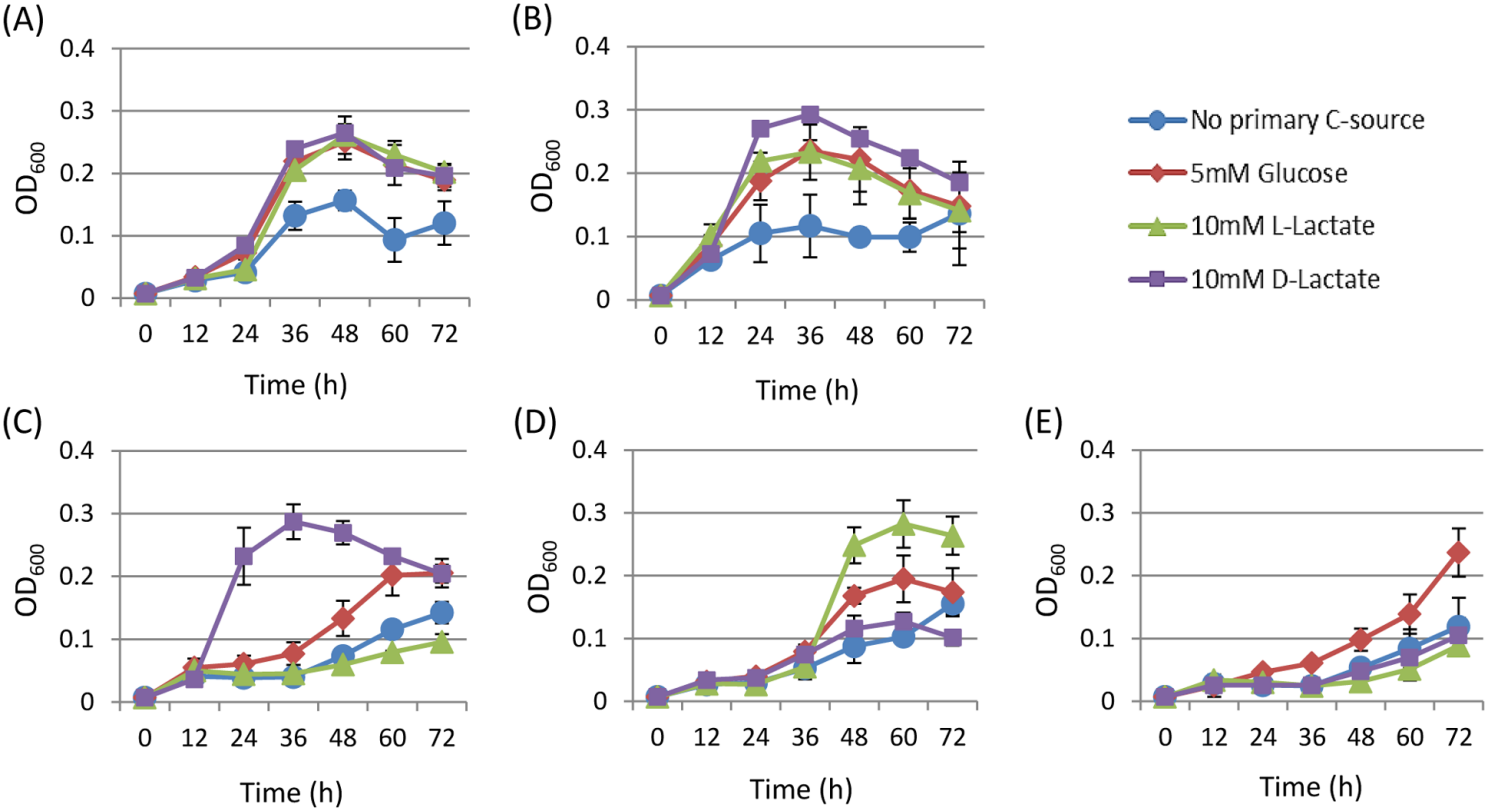
Growths of *H. pylori 26695* wild type (A), the Δ140 mutant (B), the Δ138 mutant (C), the Δ1222 mutant (D), and the Δ138/Δ1222 mutant (E) cultured in PP medium with/without a particular carbon source; no primary carbon source (blue circle), 5 mM D-glucose (red diamond), 10 mM L-lactate (green triangle), 10 mM D-lactate (purple square). The cell growth was monitored by measuring the optical density at 600± SD of three independent experiments.

To investigate the effect of primary carbon source on the growth of *H. pylori*, we cultured 26695 wild-type (WT) strain in conditioned medium with/without different carbon sources (BB, BBG, BBL, or BBD, see Materials and Methods). However, none of the carbon-supplemented media (BBG, BBL, and BBD) improved bacterial growth compared to basal medium (BB). We hypothesized that carbon supplied by the basal components such as peptone, yeast extract, and/or FBS was sufficient so that *H. pylori* 26695 growth was independent of the additional carbon source. Therefore, a minimal conditioned medium (PP, see Materials and Methods) was prepared in order to minimize the effects of the basal composition to allow the potential benefits of additional carbon sources to be identified. When cultured in PP, 26695 WT strain showed relatively weak growth rate, which was further improved in the addition of 5 mM glucose (PPG), 10 mM L-lactate (PPL), or 10 mM D-lactate (PPD), suggesting that *H. pylori* 26695 could utilize D- and L-lactate as well as glucose (Fig. 2A). Similarly, the Δ140 mutant showed improved growth with glucose and D- and L-lactate but retained relatively higher growth rates compared to the WT strain (Fig. 2B). The ability to grow with L-lactate was significantly decreased in the Δ138 mutant. Interestingly, the growth of this mutant was more favorable with D-lactate than with glucose (Fig. 2C). In contrast, the Δ1222 mutant grew relatively better with L-lactate, but failed to grow with D-lactate (Fig. 2D). When the two sets of genes (*hp0137–0139* and *hp1222*) were disrupted, the Δ138/Δ1222 mutant failed to grow with either D- or L-lactate (Fig. 2E).

### D- and L-lactate uptake is partially blocked by Δhp0140–0141, but it is still permeable to *H. pylori* cells

**Figure 3:**
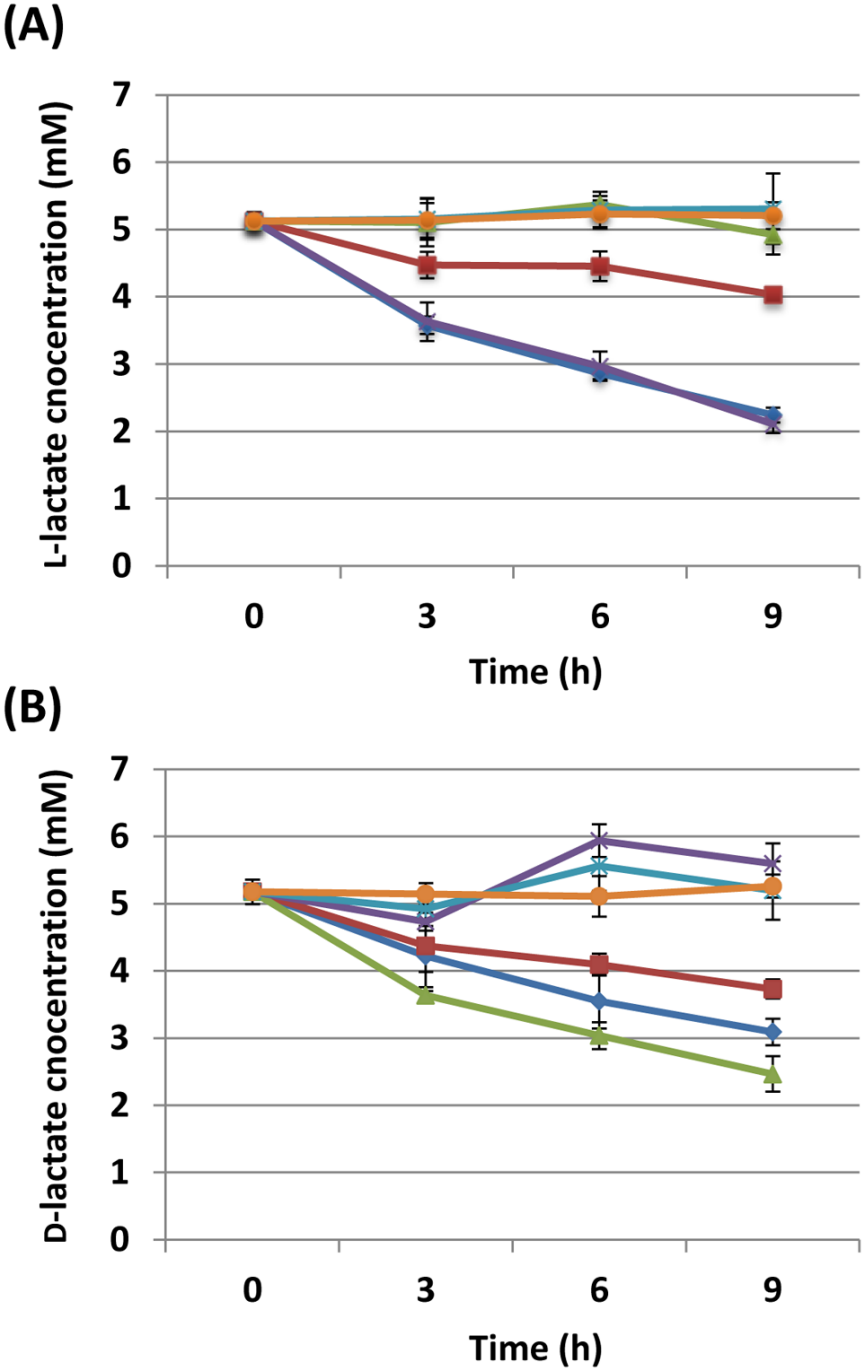
Concentrations of L-lactate (A) and D-lactate (B) in culture supernatants of *H. pylori* 26695 wild type and isogenic mutants. Exponentially growing 26695 wild type (blue), the Δ140 mutant (red), the Δ138 mutant (green), the Δ1222 mutant (purple), the Δ138/Δ1222 mutant (aqua), as well as blank medium (orange) were incubated with 5 mM L- or D-lactate, and lactate concentrations were determined by biochemical assay kits as in Materials and Methods. The data points and error bars represent the means ± SD of three independent experiments.

To confirm lactate importation/consumption by the mutants, we measured whether there was a shift of D- and L-lactate concentrations following incubation with exponentially growing *H. pylori* cells. As shown in Fig. 3A, the L-lactate concentration in supernatants was steadily reduced by 26695 WT strain and by the Δ1222 mutant in a similar manner. This consumption of L-lactate was, however, abolished in both the Δ138 mutant and the Δ138/Δ1222 double mutant, which is consistent with the observation of these mutants have impaired in growth with L-lactate. As expected, the reduction of L-lactate concentration was slower in the Δ140 mutant that lacks the putative lactate permease gene (*lctP*; *hp0140–0141*); however, the uptake of L-lactate was not totally blocked in this mutant which may explain the growth of this mutant in the presence of L-lactate as a primary carbon source. In a similar manner, the Δ140 mutant moderately reduced the concentration of D-lactate, but less than observed with the 26695 WT strain (Fig. 3B) which we interpret as D-lactate being partially blocked by Δ*hp0140–0141*. The Δ138 mutant consumed D-lactate faster than the WT strain providing clear evidence for enhanced growth of this mutant with D-lactate.

### *H. pylori* cell extract exhibited D-iLDH but not L-iLDH

**Figure 4:**
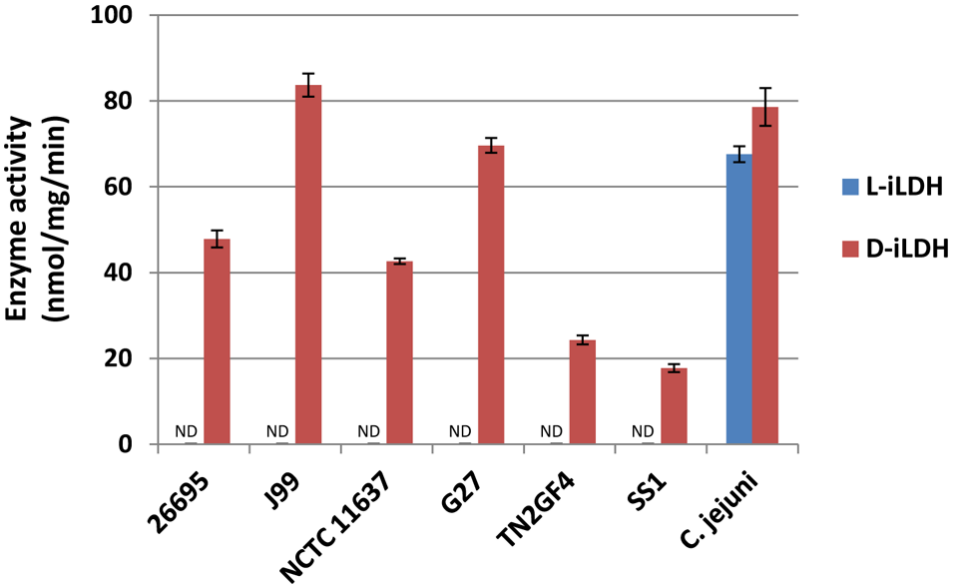
NAD-independent D- and L-lactate dehydrogenase (D- and L-iLDH) activities in cell extracts of *H. pylori* strains 26695, J99, NCTC 11637, G27, TN2GF4, SS1, and *C. jejuni* ATCC 33292. The activity was measured in cell extracts by a coupled colorimetric assay using 5- or L-lactate as an electron donor and a mixture of artificial acceptors, MTT and PMS. The activity in the first 30 min was spectrophotometrically monitored at 570 nm, and was normalized by the total protein concentration in cell extract. All the data represent the means ± SD of three independent experiments.

To provide biochemical evidence for the lactate utilizing activity, we measured D- and L-iLDH activities in cell extracts of 26695 WT strain using PMS and MTT as artificial electron acceptors. D-iLDH activity but not L-iLDH activity was detected in the first 30 minutes of the reaction. We measured D- and L-iLDH activities of 5 other *H. pylori* strains as well as *C. jejuni* ATCC 33292 to confirm the validity of the experiment. *C. jejuni*, another human gastrointestinal pathogen, shares a high degree of phylogenetic similarity with *H. pylori* and is known to exhibit both D- and L-iLDH activities [17] and was used as the positive control in the assay. None of the *H. pylori* strains demonststrated L-iLDH activity despite extending the period of 120 minutes. In contrast, all of these strains exhibited various levels of D-iLDH activities within the first 30 minutes. Moreover, both D- and L-iLDH activities were detected in cell extract of *C. jejuni* cultured under exactly the same condition as the *H. pylori* strains (Fig. 4).

### HP0137–0139 play a main role in L-nLDH activity, whereas HP1222 contributes to both the D-iLDH and L-nLDH activities

**Figure 5:**
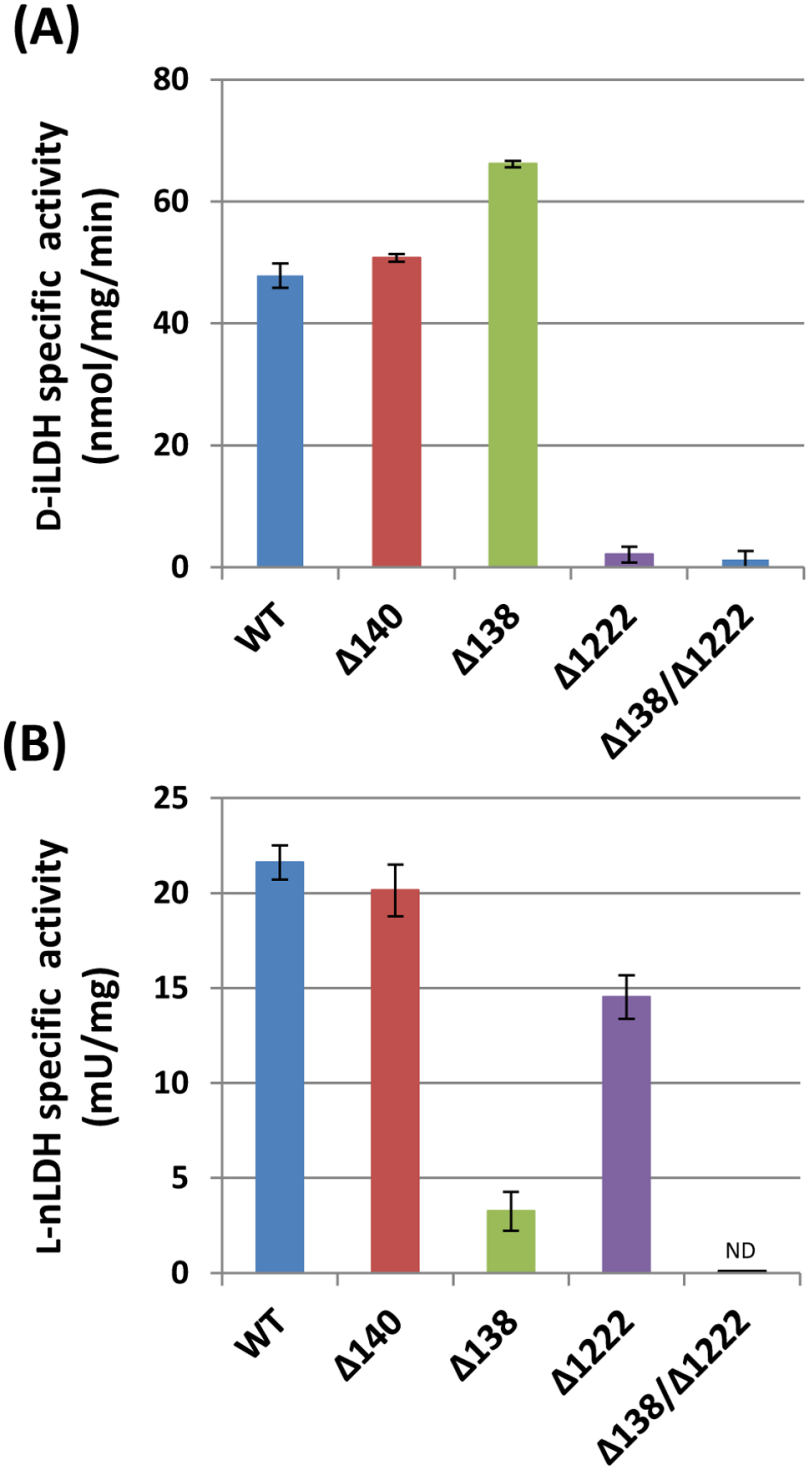
NAD-independent D-lactate dehydrogenase (D-iLDH) and NAD-dependent L-lactate dehydrogenase (L-nLDH) activities in cell extracts of *H. pylori* 26695 wild type and isogenic mutants. (A) D-iLDH activity was measured as described above. (B) L-nLDH activity was measured by monitoring the reduction of NAD to NADH, arising from the conversion of L-lactate to pyruvate, which was spectrophotometrically detected at 450 nm, and was calibrated based on enclosed NADH standards. All the data represent the means ± SD of three independent experiments, and one unit of the activity is defined as the amount of enzyme that catalyzes the conversion of lactate into pyruvate to generate 1.0 µmole of NADH per minute at 37°C.

The MTT assay was used with cell extracts of 26695 mutants in order to verify the contributions of candidate genes to D-iLDH activity. Under the conditions described above, a dramatic decrease in D-iLDH activity (>95%) was observed in both the Δ1222 single mutant and the Δ138/Δ1222 double mutant compared to the WT strain (Fig. 5A). These confirm the function of HP1222 as a D-iLDH of *H. pylori*. Almost the same level of D-iLDH activity was observed in the Δ1222 and Δ140 mutants, whereas the D-iLDH activity was slightly increased in theΔ138 mutant, which was in accordance with the results of the growth experiments and lactate uptake assay using this mutant (Fig. 2C and Fig. 3B).

Since no L-iLDH activity was detected in the cell extract of the 26695 WT strain, we assessed L-nLDH activity in the same cell extract. As described in Methods, an increase of NADH, which is proportional to the amount of NAD reduced in the conversion of L-lactate into pyruvate, was detected. In this assay, the cell extract of 26695 WT strain exhibited activities of 21.6±0.9 mU mg protein^−1^. A nearly 7-fold decrease in the activity was observed using the Δ138 mutant compared to the WT strain. The L-nLDH activity was unaffected in the Δ140 mutant, while the activity was partially decreased in the Δ1222 mutant; moreover, the L-nLDH activity decreased to an undetectable level in the Δ138/Δ1222 double mutant (Fig. 5B).

### Lactate utilization genes are constitutively expressed in *H. pylori*

**Figure 6:**
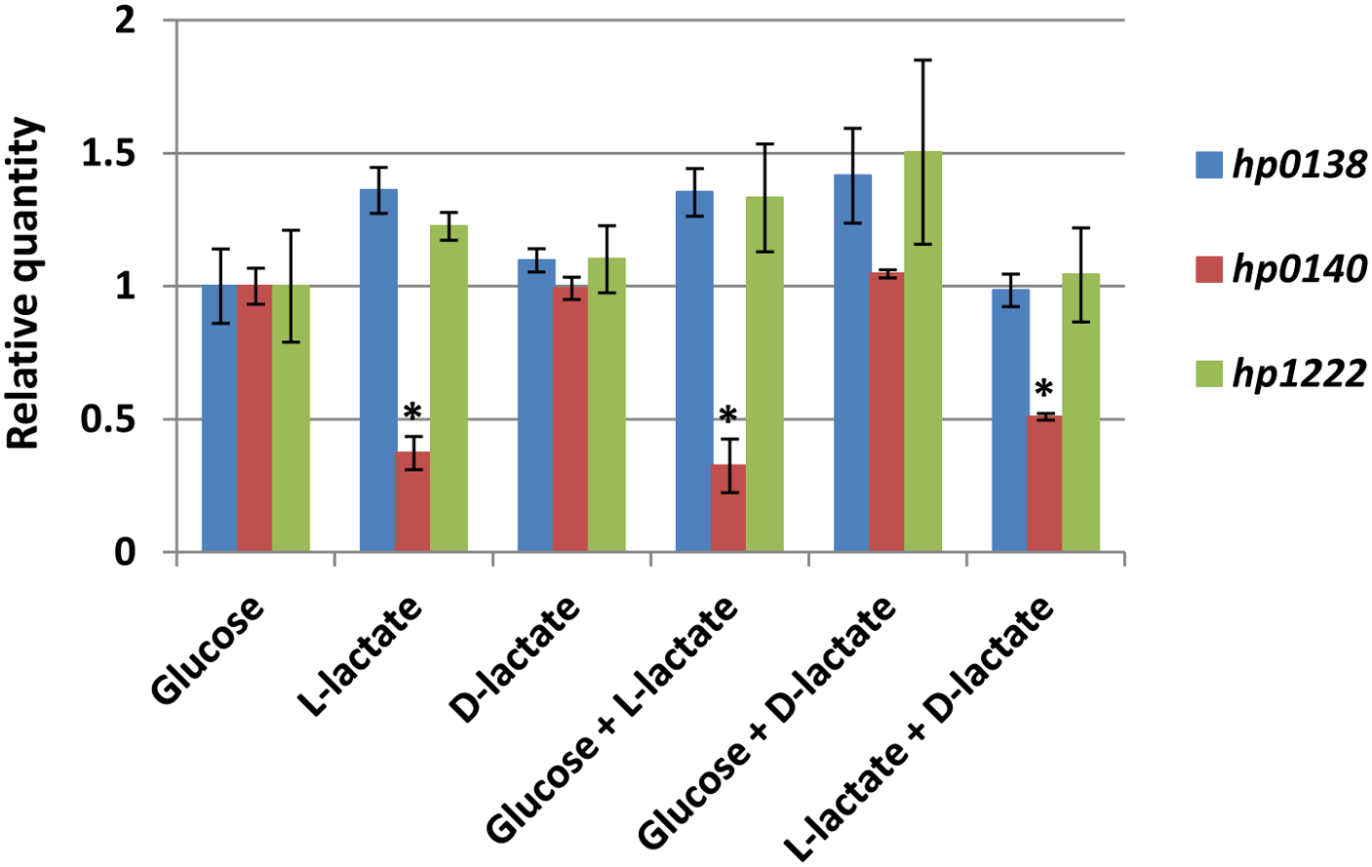
The relative gene expressions of *hp0138*, *hp0140*, and *hp1222* in *H. pylori* 26695 wild type grown in a variety of carbon sources (5 mM glucose, 10 mM L-lactate, 10 mM D-lactate, 2.5 mM glucose+5 mM L-lactate, 2.5 mM glucose+5 mM D-lactate, or 5 mM L-lactate+5 mM D-lactate). Real-time quantitative PCRs were performed in 7300 Real-Time PCR System (Applied Biosystems) using Taqman-designed primers and probes (Life technologies), and the data were analyzed by comparative Ct (ΔΔCt) method with 16S rRNA as the reference gene. All the quantifications were performed in triplicate and were shown as the means ± SD.

To analyze whether lactate was involved in the regulation of lactate utilization genes in *H. pylori*, we quantified the expression levels of *hp0138*, *hp0140*, and *hp1222* in the 26695 WT strain that was cultured in BB medium with a variety of carbon sources [5 mM D-glucose; 10 mM L-lactate; 10 mM D-lactate; 2.5 mM D-glucose+5 mM L-lactate; 2.5 mM D-glucose+5 mM D-lactate; 5 mM L-lactate+5 mM D-lactate]. Despite the carbon source(s) supplemented, 26695 WT strain showed similar patterns of growth curves (data not shown). Total RNAs were extracted from exponentially growing cells in each culture batch and the *hp0138* and *hp1222* genes were constitutively expressed irrespective of the carbon source(s) added. In contrast, the expression of *hp0140* was not dramatically, but was significantly (P<0.001) suppressed in the presence of L-lactate (Fig. 6). Moreover, the suppressed expression of *hp0140* was only present with L-lactate but not with D-lactate.

## Discussion

Studies of *H. pylori* energy metabolism, including substrate oxidation and primary dehydrogenase, has been studied [8] and although some metabolic studies have demonstrated the oxidation of D- and L-lactate in intact *H. pylori* cells [21], [22] the corresponding genes and their detailed function remain unknown and no isogenic mutants have been previously described. Here, we report the construction of isogenic mutants of the candidate genes Δ*hp0137–0139*, Δ*hp0140–0141*, and Δ*hp1222* in parental *H. pylori* strain 26695. We show that *hp0140–0141* encode a lactate permease (LctP) which imports both D- and L-lactate. However, moderate uptake of D- and L-lactate was observed in the Δ140 mutant suggesting the presence of an alternative route(s) for lactate transport in *H. pylori*. In *E. coli*, the *lctP*-encoding permease protein shares a high degree of similarity to the product of *yghK* (*glcA*) gene in glycolate utilization operon. Because of structural and functional similarities, both LctP and GlcA are able to transport glycolate as well as D- and L-lactate in a proton motive force dependent manner [23], [24]. This mechanism is likely not applicable to *H. pylori* as no GlcA orthologue has been found in its genome; conversely, in *H. pylori* LctP could function in glycolate transport as glycolate oxidase is present in this bacterium (GlcD; HP0509/JHP459) [7], [9]. The Δ140 mutant grew well in PPL or PPD media probably because this alternative route(s) provided sufficient amount of lactate for the cell growth. Considering the fact that we did not recognize any growth advantage of *H. pylori* 26695 WT in other conditional media (BBG, BBL, BBD in comparison to BB), *H. pylori* may not require a large amount of carbon source if its metabolism is working properly. According to the genetic organizations of other *Helicobacter* sp., the presence of two parallel *lctP* genes (*lctP1* and *lctP2*) may be a unique feature of *H. pylori* as only one *lctP* gene is recognized in other *Helicobacter* sp. (GenBank).

Inactivation of *hp0137–0139* genes resulted in the mutant being unable to consume and thus grow on L-lactate. In general, lactate transport across cell membrane is bidirectional, and it contributes to the balance of extracellular and intracellular lactate concentrations. As the Δ138 mutant is unable to consume L-lactate, there was apparently no decrease of extracellular lactate. Interestingly, the Δ138 mutant consumed D-lactate faster than the WT strain. This was consistent with the increased D-iLDH activity detected in its cell extract compared to the WT strain. This suggests the possibility of an antagonistic effect between D- and L-lactate or between the functions of enzymes (D- and L-LDH) either in a direct or indirect manner. Given that LldEFG (LutABC) orthologues are known to oxidize L-lactate in an NAD-independent manner (L-iLDHs) [13], [17], it was unexpected that we did not detect L-iLDH activities in any of the *H. pylori* strains tested. We enriched the concentration of protein extracts, prolonged the reaction period, and/or enlarged the number of strains tested in order to many any improvement on this method; however, only D-iLDH activity but not L-iLDH activity was detected from *H. pylori* strains. In other bacteria, *lldEFG*-encoding L-iLDH is sometimes inactive and another L-iLDH or L-nLDH catalyze the oxidation of L-lactate [25], [26]. Thus, we measured NAD-dependent LDH activity in *H. pylori*, which catalyzes the conversion of L-lactate to pyruvate, by reducing NAD to NADH. The results clearly demonstrated the contribution of HP0137–0139 to the L-nLDH activity in this bacterium. We currently have no clear explanation for this cofactor dependency as none of the HP0137–0139 proteins is predicted to contain a NAD-binding domain. However, based on our experimental data, we conclude that HP0137–0139 contributes to the oxidation of L-lactate with NAD as a potential mediator. NAD-dependent LDHs are generally called fermentative LDHs and known to convert pyruvate to lactate in glycolysis; in addition, many of these enzymes catalyze reversible reactions at the same time [1]. Previous metabolic studies of *H. pylori* have revealed that this bacterium is capable of producing lactate as one of the end products of glucose and pyruvate metabolism [27]–[29]. Because no other candidate for L-LDH has been found in the *H. pylori* genome, it is possible that HP0137–0139 also contributes to the production of lactate, although this remains to be proven.

HP1222, initially annotated as Dld, was re-categorized as Dld-II in recent study based on the presence of an additional C-terminal 4Fe-4S-binding domain that is not present in classic Dld [13]. Although Dld-II orthologues are assumed to oxidize D-lactate, the stereoisomer specificity of this enzyme is somewhat controversial as the orthologue in *C. jejuni* (Cj1585c) was identified as L-iLDH but not D-iLDH [17]. Cj1585c is one of the highly similar orthologues of HP1222 sharing ca. 50% identity with each other. This similarity may explain the decreased L-LDH activity observed in the Δ1222 mutant. In either case, HP1222 is clearly dedicated to the D-lactate utilization in *H. pylori*, and is less important in its L-lactate utilization as the mutant showed sufficient growth in the L-lactate-dependent medium. Unlike the other bacteria that have a lactate-specific transcriptional regulator, the expression of both *hp1222* and *hp0137–0139* in *H. pylori* are apparently constitutive and not inducible by lactate. A previous study using a microarray approach found *hp1222* to be up-regulated in acidic conditions [30], although this gene was not involved in the regulation under low-pH condition in another microarray study [31]. None of the *hp0137–0139* genes were detected in either two microarray study. However *hp0140* gene expression was slightly suppressed in the presence of L-lactate but not D-lactate. Although the details remain unclear, there may be a sensor-like mechanism present in *H. pylori* that adjusts the concentration of intracellular and extracellular lactate by modulating the expression of the permease. It was previously reported that *hp0140* was up-regulated under iron starvation condition [32] which is consistent with the multiple 4Fe-4S binding cluster domains involved in HP0137–0139 and HP1222 structures.

Lactate utilization is associated with the pathogenicity of some bacteria such as *Neisseria gonorrhoeae*, *N. meningitides*, and *Haemophilus influenzae*. In those bacteria, lactate is incorporated into the energy cycle as well as the synthetic pathway of some pathogenic determinants such as lipopolysaccharide and polysialic acid capsule [33], [34]. The inability to utilize lactate significantly decreases the abilities of those pathogenic bacteria to colonize and survive in vivo [35]–[37]. It is noteworthy that a previous study using transposon mutagenesis confirmed *hp0137* as one of the genes that contribute to the colonization of *H. pylori* in mouse stomach [38]. At that time HP0137 was described as an uncharacterized hypothetical protein.

In the current study, we demonstrated that *H. pylori* can utilize both D- and L-lactate which involves the gene products of *hp0140–0141* (*lctP*), *hp0137–0139* (*lldEFG*), and *hp1222* (*dld-II*). To explore how this ability affects the pathogenicity of *H. pylori* in vivo, an animal infection model using currently constructed *H. pylori* mutants is under investigation.

## Supporting Information

Table S1 Primers used in this study.
(DOCX)
