# Supplementary material for: Identification of the Genes That Contribute to Lactate Utilization in Helicobacter pylori
Source: PLoS One. 2014 Jul 31;9(7):e103506. doi: 10.1371/journal.pone.0103506 (PMC4117512; doi:10.1371/journal.pone.0103506)
Supplement: Table S1 — Primers used in this study. (DOCX) [file pone.0103506.s001.docx]

Table S1. Primers used in this study

| Primer | Nucleotide sequence* | Use application |
| --- | --- | --- |
| Lct1-F | 5’-CTTTTTCCAATTCAATGAGTTCCAC-3’ | KO plasmid construction |
| Lct2-R | 5’-GGCTTGGTTATTATTCAAGGGC-3’ | KO plasmid construction |
| Lct3-F | 5’-TAGATTTAGCAGGGTTTAACCC-3’ | KO plasmid construction |
| Lct4-R | 5’-GCCTAATGGGTCATAGACTTG-3’ | KO plasmid construction |
| hp139-F0 | 5’-GCCTTCTTTG*T*AAGTCAATTTCTTTGC-3’ | KO plasmid construction |
| hp137-R0 | 5’-GAGTAAAGAGCTTATTTTAAAGCGC-3’ | KO plasmid construction |
| hp137-F0 | 5’-CAATCTAGTTCTAAAAAATGTCAATATGG-3’ | KO plasmid construction |
| hp139-R0 | 5’-TAAAGCCCAAGTCTTGAGGC-3’ | KO plasmid construction |
| hp1222-F0 | 5’-CGTGTGGAAGAAAATTATCATGC-3’ | KO plasmid construction |
| hp1222-R0 | 5’-TAAAGCGTGCAAGCATCCAC-3’ | KO plasmid construction |
| hp1222-F1 | 5’-CCCAAAAACAACGCCTATCC-3’ | KO plasmid construction |
| hp1222-R1 | 5’-GACTGATGATTTCAATAGGGTCT-3’ | KO plasmid construction |
| hp138-qF | 5’-GAAAAGAGGGCGATTTAGAC-3’ | RT-PCR |
| hp138-qR | 5’-CATGAAAGAGCGCGATAATAC-3’ | RT-PCR |
| hp140-qF | 5’-CTTTCGCCTATTGCGCTC-3’ | RT-PCR |
| hp140-qR | 5’-TCGCGCTCACCATTTGAG-3’ | RT-PCR |
| hp1222-qF | 5’-CAAATCAGCAGTGATCCTAG-3’ | RT-PCR |
| hp1222-qR | 5’-TCTTGACTGAAGCAGATGTC-3’ | RT-PCR |
| Hp16S-F | 5’-CTGGAGAGACTAAGCCCTCC-3’ | RT-PCR |
| Hp16S-R | 5’-ATTACTGACGCTGATTGTGC-3’ | RT-PCR |

*An underlined italic letter indicates a substituted nucleotide.
